# Haptoglobin Genotypes in Hidradenitis Suppurativa: Haptoglobin 2–2 Genotype Is Associated With Familial Hidradenitis Suppurativa and More Flares

**DOI:** 10.1111/ijd.17705

**Published:** 2025-02-28

**Authors:** Nessr Abu Rached, Marina Skrygan, Lennart Ocker, Yannik Haven, Daniel Myszkowski, Eggert Stockfleth, Falk G. Bechara

**Affiliations:** ^1^ International Centre for Hidradenitis Suppurativa/Acne Inversa (ICH), Department of Dermatology, Venereology and Allergology Ruhr‐University Bochum Bochum Germany; ^2^ Skin Cancer Center, Department of Dermatology, Venereology and Allergology Ruhr‐University Bochum Bochum Germany

**Keywords:** haptoglobin, haptoglobin genotype, hidradenitis suppurativa, inflammation

## Abstract

**Background:**

Haptoglobin (Hp) is an acute‐phase protein and an independent marker for hidradenitis suppurativa (HS) severity. The different Hp genotypes (Hp 1–1, Hp 1–2, and Hp 2–2) differ in their antioxidant and anti‐inflammatory functions. Hp genotypes have never been investigated in HS patients.

**Objective:**

Our aim was to characterize and determine the frequency of Hp genotypes in HS patients. Additionally, the characteristics of the different Hp genotypes are analyzed for differences in personal, disease‐specific, and laboratory parameters.

**Methods:**

Genomic DNA was extracted from peripheral blood leukocytes using the Maxwell RSC Blood DNA Kit. Polymerase chain reaction (PCR) products were separated and analyzed using 0.7% agarose gels for genotyping. The chi‐square test, Kruskal–Wallis test, and/or post hoc analysis with the Dunn–Bonferroni test were used to determine differences between the different genotypes and HS characteristics. We compared the genotype distribution in HS with 69 randomly selected, unmatched, and healthy control patients.

**Results:**

A total of 72 patients with HS were included in this study, including 29 women (40.3%) and 43 men (59.7%). A positive family history of HS was reported by 29.2% of patients (*n* = 21). Hp genotype 1–1 was present in 20.8%, Hp 1–2 in 33.3%, and Hp 2–2 in 45.8% of HS patients. The control group had a similar Hp distribution with 18.8%, 37.7%, and 43.5%, respectively. A positive family history of HS was significantly more frequent in Hp 2–2 (42.4%) compared to Hp 1–1 (6.7%) and Hp 1–2 (25%) (*p* = 0.035). The number of HS flares in the last 4 weeks was significantly higher in Hp 2–2 patients compared to Hp 1–2 patients (*p* = 0.006).

**Conclusion:**

Hp genotype 2–2 and the Hp2 allele were significantly more associated with familial HS than the other genotypes. In addition, HS patients with genotypes 2–2 had more frequent flare‐ups than those with the different genotypes.

## 1 Introduction

Hidradenitis suppurativa (HS) is a chronic, inflammatory skin disease manifested by recurrent painful abscesses, nodules, and fistulas that often occur with many comorbidities [1]. For example, common comorbidities of HS include metabolic syndrome, obesity, diabetes mellitus, inflammatory bowel diseases, psoriasis vulgaris, and autoimmune diseases [2, 3, 4, 5, 6, 7]. These associations between HS and comorbidities indicate that the biological mechanisms responsible for both HS and concomitant comorbidities are not yet understood. It is also not clear why only a subgroup of HS patients develops severe metabolic complications. HS is characterized by chronic inflammation, as evidenced by laboratory and clinical findings [8, 9]. Haptoglobin (Hp) is a new serological marker indicating HS's severity and metabolic risk [10]. Haptoglobin is a protein in the blood that plays an essential role in binding free hemoglobin and thus influences inflammatory processes and oxidative stress. Haptoglobin has two alleles, Hp1 and Hp2, resulting in three genotypes: Hp1‐1, 1–2, and 2–2 [11, 12]. Hp 1–1 is known for its efficient ability to bind hemoglobin and reduce oxidative stress [13]. Hp 2–1 and Hp 2–2 show a lower binding affinity to hemoglobin and are less effective at reducing oxidative stress. The different genotypes are associated with various comorbidities. For example, Shi et al. showed that patients with the Hp2–2 genotype had a significantly higher risk of type 2 diabetes than those with other genotypes [14]. Hp genotypes were never analyzed in HS patients.

In this study, we aimed to investigate the haptoglobin genotypes of patients with HS and to compare them with a non‐HS group. In this study, we aimed to examine the haptoglobin genotypes of HS patients and compare them with a non‐HS group.

## 2 Patient and Methods

### 2.1 Design, Setting, and Patients

We collected personal (sex, age, age of HS onset, smoking status, and comorbidities) and disease‐related data [HS disease severity, Dermatology Life Quality Index (DLQI) [15], number of flare‐ups, pain, and affected regions]. HS disease severity was assessed using the SAHS score, mHSS score, and the Hurley system [16, 17, 18, 19, 20]. The data and patients were obtained from a prospective study [10]. The laboratory parameters collected included the number of leukocytes, platelets, neutrophil granulocytes, lymphocytes, eosinophil granulocytes, basophil granulocytes, C‐reactive protein (CRP), haptoglobin, ferritin, and transferrin. According to Dessau, only HS patients with a confirmed diagnosis and complete data were included [21]. To compare the genotype distribution in HS, the genotypes of 69 randomly selected, unmatched, and healthy control patients were determined.

### 2.2 Determination of Haptoglobin Genotypes

Genomic DNA was extracted from peripheral blood leukocytes using the Maxwell RSC Blood DNA Kit as recommended by the supplier (Promega). Hp genotyping was performed using the polymerase chain reaction (PCR)‐based method, according to Koch et al. (2002). Three sets of primers were used to amplify an Hp 1 allele‐specific sequence and an Hp 2 allele‐specific sequence [22]. The allele‐specific primers were synthesized by TibMolBiol (Berlin, DE). Depending on the genotype represented by the template DNA, an Hp1‐specific product and/or an Hp2‐specific product was generated in PCRs. PCR products were separated into 0.7% agarose gels for genotype assignment and analyzed. Genotype determinations were performed without knowledge of the phenotyping results.

### 2.3 Statistical Analysis

All results and data are presented as numbers (percentages) and medians [interquartile ranges (IQRs)]. To analyze baseline patient data and disease‐specific data, we used descriptive statistics. Data were analyzed using IBM SPSS Statistics version 29.0 (IBM Corporation). A significance level of *p* < 0.05 was used. The chi‐squared test, Kruskal–Wallis test, and/or post hoc analysis with the Dunn–Bonferroni test were used to determine differences between the different genotypes.

## 3 Results

### 3.1 Disease‐Specific and Personal Patient Characteristics

A total of 72 patients with HS were included in the study, including 29 women (40.3%) and 43 men (59.7%). Patients' mean age was 40.4 years (SD: ±11.2), with a median age of onset of HS of 21 years (range: 18–31.3). Mean body mass index (BMI) was 32.2 kg/m^2^ (±6.8), with most patients classified as obese (Table 1). A positive family history of HS was reported by 29.2% of patients (*n* = 21), while the majority (70.8%; *n* = 51) had no family history. Smoking was prevalent, with 63.9% active smokers and 33.3% non‐smokers. Disease severity was assessed using the Hurley staging system, with the majority of patients in Hurley stage II (62.5%), followed by stage III (33.3%) and stage I (4.2%). The median SAHS score was 7 (IQR: 5–9), the median modified HS Score (mHSS) was 36 (IQR: 20.5–75.5), and the DLQI had a median score of 13 (IQR: 9–20), reflecting a significant impact on quality of life. Patients reported a median of 1 flare (IQR: 0–2) in the past 4 weeks, with a median visual analogue scale pain score of 4 (IQR: 2–6). Comorbid conditions were common, with 56.9% of patients being obese, 27.8% having hypertension, and 15.3% having diabetes. The inguinal (62.5%) and axillary (61.1%) regions were the most affected areas. The distribution of haptoglobin genotypes among HS patients was as follows: Hp 1–1 in 20.8% (*n* = 15), Hp 1–2 in 33.3% (*n* = 24), and Hp 2–2 in 45.8% (*n* = 33). The control group showed a similar Hp distribution with 18.8% (*n* = 13), 37.7% (*n* = 26), and 43.5% (*n* = 30), respectively.

**TABLE 1:** Personal characteristics and disease‐specific characteristics of patients with HS.^a^

Parameter	Value
Sex, *n* (%)	
Female	29 (40.3)
Male	43 (59.7)
Age, mean (±SD), y	40.4 (±11.2)
Age of HS onset, median (range), y	21 (18–31.3)
BMI, mean (±SD), kg/m^2^	32.2 (±6.8)
Family history of HS, *n* (%)	
Positive	21 (29.2)
Negative	51 (70.8)
Smoking status, *n* (%)	
Smokers	46 (63.9)
Ex‐smokers	2 (2.8)
Non‐smokers	24 (33.3)
Hurley stage, *n* (%)	
Hurley I	3 (4.2)
Hurley II	45 (62.5)
Hurley III	24 (33.3)
SAHS, median (IQR)	7 (5–9)
mHSS, median (IQR)	36 (20.5–75.5)
DLQI, median (IQR)	13 (9–20)
Number of flare‐ups in the last 4 weeks, median (IQR)	1 (0–2)
Pain according to the visual analogue scale, median (IQR)	4 (2–6)
Number of affected regions, *n* (%)	2.5 (2–4)
Haptoglobin genotype, *n* (%)	
Hp 1–1 in the HS group^a^	15 (20.8)
Hp 1–1 in the control group^b^	13 (18.8)
Hp 1–2 in the HS group^a^	24 (33.3)
Hp 1–2 in the control group^b^	26 (37.7)
Hp 2–2 in the HS group^a^	33 (45.8)
Hp 2–2 in the control group^b^	30 (43.5)

Abbreviations: BMI, body mass index; DLQI, Dermatology Life Quality Index; Hp, haptoglobin; IQR, interquartile range; mHSS, modified Hidradenitis Suppurativa Score; *n*, absolute number of patients; SAHS, Severity Assessment of Hidradenitis Suppurativa Score; SD, standard deviation; y, years.

^a^ Total number of HS patients: 72.

^b^ Total number of control patients: 69.

### 3.2 Differences Between Hp1‐1, 1–2, and 2–2 Genotypes

**FIGURE 1:**
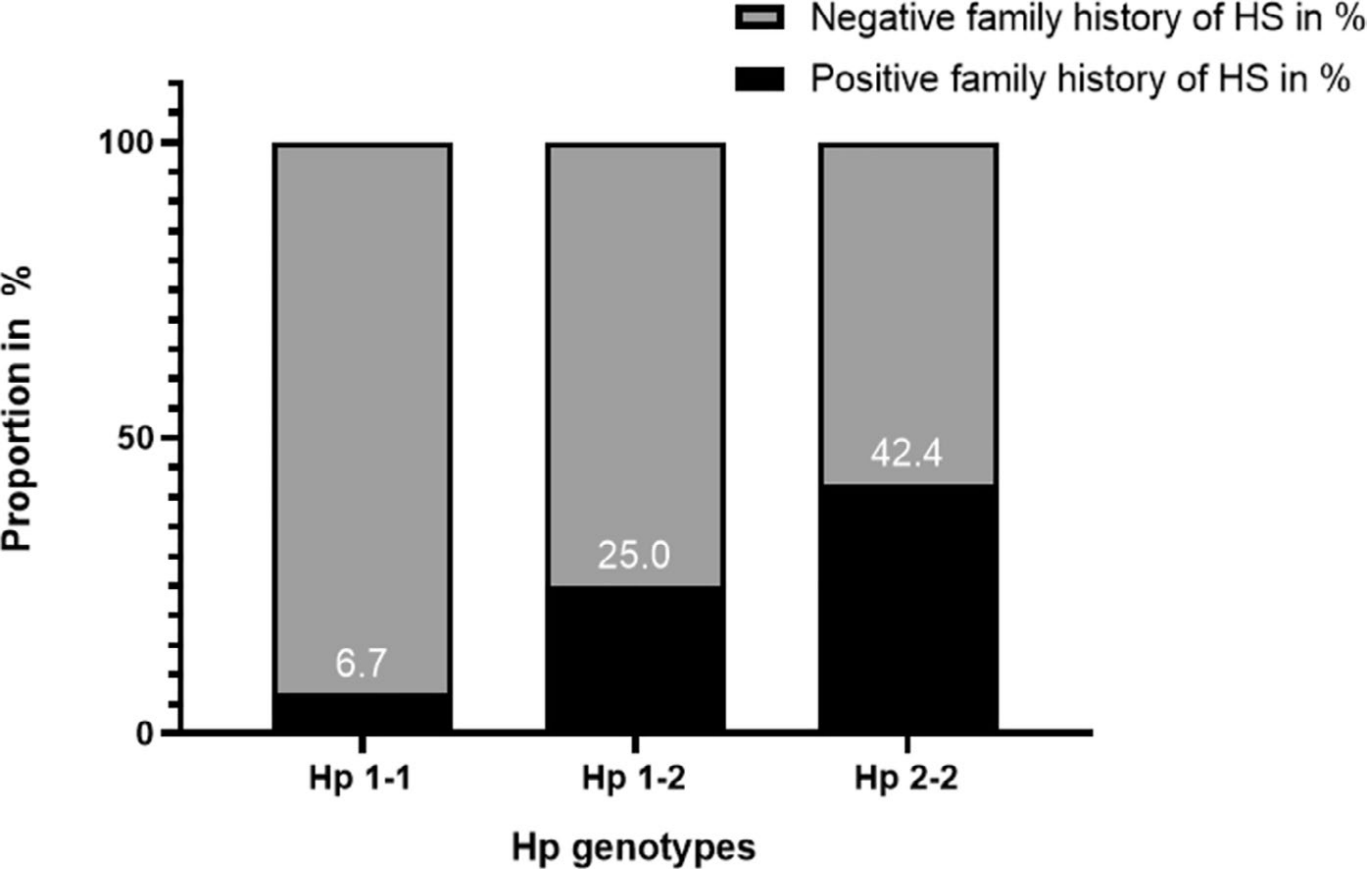
Percentage of positive family history for HS sorted by haptoglobin genotypes 1–1, 1–2, and 2–2; black, positive family history for HS; gray, negative family history for HS.

Hp genotypes (Hp 1–1, Hp 1–2, and Hp 2–2) were statistically analyzed using chi‐square, Kruskal–Wallis, and post hoc Dunn–Bonferroni tests (Table 2). A positive family history of HS was significantly more common in Hp 2–2 (42.4%; *n* = 14) compared to Hp 1–1 (6.7%; *n* = 1) and Hp 1–2 (25%; *n* = 6) (*p* = 0.035; Figure 1). No significant differences between genotypes were observed for most personal parameters, including age at HS onset (*p* = 0.4), body mass index (BMI) (*p* = 0.8), and comorbidities such as obesity (*p* = 0.5) and diabetes (*p* > 0.9).

**TABLE 2:** Person‐specific, HS‐specific, and laboratory characteristics of the genotypes Hp 1–1, 1–2, and 2–2 using the chi‐square test, Kruskal–Wallis test, and/or post hoc analysis by Dunn–Bonferroni test.

Parameter	Genotype Hp 1–1 (Hp1 allele)	Genotype Hp 1–2	Genotype Hp 2–2 (Hp2 allele)	*p* ^e^	Post hoc analysis^f^
Personal‐specific parameter					
Absolute number (%)^a^	15 (20.8)	24 (33.3)	33 (45.8)	NA	NA
Male^b^	9 (60)	14 (58.3)	20 (60.6)	> 0.9	NA
Age of HS onset^c^	28 (18–33.5)	25 (18.8–34.5)	20 (18–29)	0.4	—
BMI^c^	32.3 (27.2–36.7)	31.5 (26–36.2)	32.7 (27–38.1)	0.8	—
Obesity^b^	7 (46.7)	13 (54.2)	22 (63.6)	0.5	NA
Hypertension^b^	6 (40)	5 (20.8)	9 (27.3)	0.4	NA
Diabetes mellitus^b^	2 (13.3)	4 (16.7)	6 (15.2)	> 0.9	NA
Acne vulgaris/conglobate^b^	0 (0)	2 (8.3)	7 (21.2)	0.09	NA
Hypothyroidism^b^	1 (6.7)	1 (4.2)	6 (18.2)	0.2	NA
HS‐specific parameter					
Positive family history of HS^b^	1 (6.7)	6 (25)	14 (42.4)	0.035*	NA
Hurley III^b^	5 (33.3)	8 (33.3)	11 (33.3)	0.4	NA
SAHS^c^	8 (5.5–10)	6.5 (5–9)	8 (6–10)	0.3	NA
mHSS^c^	42 (22.5–78)	28.5 (17–71.3)	44 (28.8–67.8)	0.3	—
DLQI^c^	14 (11–18.5)	18 (10–20)	10 (4.5–19.5)	0.4	—
Number of flare‐ups in the last 4 weeks^c^	0 (0–1)	0 (0–1)	1 (0–2)	0.015*	1–2 versus 2–2 *p* = 0.006*
Pain, according to the visual analogue scale^c^	4 (2.5–6)	5 (2.8–7)	3 (0–5)	0.08	—
Number of affected regions^c,^, ^d^	3 (2–4)	2 (1–3)	3 (2–5)	0.07	—
Biologics therapy for HS^b^	5 (33.3)	7 (29.2)	15 (45.5)	0.4	—
Laboratory parameter					
CRP^c^	6 (5–18.2)	5.7 (5–15.8)	6.3 (5–15.4)	0.9	—
Ferritin^c^	140.5 (84.5–200)	125 (54.3–170)	133 (86.6–245)	0.7	—
Transferrin^c^	292 (270.5–318.8)	279 (265–305.5)	255 (236–274)	0.02*	1–1 versus 2–2 *p* = 0.011*
Haptoglobin^c^	252 (238–296)	216 (166.3–245.2)	209.5 (156.3–272.1)	0.13	—

*Note: p* values: *significant result < 0.05; —, no signficance.

Abbreviations: BMI, body mass index; CRP, c‐reactive protein; DLQI, Dermatology Life Quality Index; HS, hidradenitis suppurativa; mHSS, Modified Hidradenitis Suppurativa Score; SAHSS, Severity Assessment of Hidradenitis Suppurativa.

^a^ Total number of HS patients: 72.

^b^ Absolute number (%).

^c^ Median (interquartile range).

^d^ Affected region of the HS.

^e^ Analysis with chi‐square test, Kruskal–Wallis test, and/or post hoc analysis by Dunn–Bonferroni test.

^f^ Post hoc analysis by Dunn–Bonferroni test.

Regarding HS‐specific parameters, the number of flares in the last 4 weeks was significantly higher in Hp 2–2 patients compared to Hp 1–2 (*p* = 0.006). No significant differences were found in disease severity scores (Hurley stage, SAHS, mHSS), quality of life (DLQI), or number of affected regions (*p* > 0.05).

Laboratory parameters, including CRP and leukocyte counts, did not show significant differences between genotypes. However, transferrin levels were significantly lower in Hp 2–2 compared to Hp 1–1 (*p* = 0.011). Haptoglobin levels tended to be lower in Hp 2–2 (*p* = 0.13), but this did not reach statistical significance.

## 4 Discussion

Haptoglobin (Hp) is an acute‐phase protein associated with HS disease severity [10]. Hp binds free hemoglobin in the blood, thus reducing oxidative stress and inflammatory processes [23, 24]. There are 2 Hp alleles in humans, resulting in three Hp genotypes (Hp 1–1, 1–2, and 2–2) [11]. Of these variants, Hp 2–2 protects less against oxidative stress [14, 25, 26]. Hp genotype modulates the balance of pro‐inflammatory and anti‐inflammatory cytokines in macrophages [27]. The reduced antioxidant function of genotype 2–2 is related to the structure of Hp 2–2. The Hp molecule is typically chemically composed of two α‐subunits and one β‐subunit. In humans, there are two types of α (weight from α1 9 kDa and α2 16 kDa) and a single type of β subunit with a weight of 45 kDa [28]. Proteins of genotype Hp 1–1 consist of α1 subunits and proteins of genotype 2–2 consist of α1 subunits [29]. α1 subunit contains a single cysteine residue, which can only form a dimer. α2 subunit contains an extra subunit from cysteine residue, so it can form a polymer and is more complex [29]. Interestingly, HS patients with genotype 2–2 had significantly more frequent flare‐ups compared to the other genotypes. This may be related to the reduced antioxidant function of Hp 2–2. Further research is needed to confirm this hypothesis. However, the Hp genotypes were not associated with the severity of HS disease. This may indicate that the Hp genotypes are not the cause of HS but may be a triggering factor and that relapses are significantly more frequent with Hp 2–2.

HS patients with elevated haptoglobin levels had an increased risk of developing metabolic complications (grade III obesity and/or diabetes mellitus) [10]. In the literature, certain comorbidities are also associated with genotypes. For example, a case–control study showed that the risk of coronary heart disease is about 4 times higher with the Hp‐2‐2 genotype than with the Hp‐1‐1 genotype [30, 31]. Levy et al. found that the haptoglobin 2–2 phenotype was 5 times more associated with cardiovascular disease in patients with diabetes mellitus than Hp 1–1 [32]. We did not find an association between cardiovascular events and HS, but the number of cardiovascular events (e.g., myocardial infarction) in the HS group is probably too small to make a conclusive evaluation. Vasudevan et al. described that Hp 2–2 is associated with chronic renal disease incidence, progression, and mortality [33]. In addition, Hp 2–2 was associated with more atherosclerosis and thicker intima‐media thickness of the carotid artery [25]. All study results show that the Hp 2–2 genotype is associated with reduced oxidative function. Our study found no association between the other most common comorbidities and Hp genotypes in HS patients.

Koch et al. described an Hp genotype distribution of 14.5% for Hp 1–1, 48.2% for Hp 1–2, and 37.3% for Hp 2–2 in Germany [22]. Another study by Adams et al. reported a frequency of 13.9% for Hp 1–1, 41.8% for Hp 1–2, and 44.3% for Hp 2–2 [34]. These Hp distributions are similar to those of our HS group and our control group. This demonstrates that the Hp genotypes in HS patients are not shifted to one genotype and are not the cause of the disease.

A limitation of the study is the limited sample size. Another limitation is the lack of long‐term data on cardiovascular risk over several years, which limits the influence and significance of Hp genotypes on comorbidities. The analysis focuses exclusively on the haptoglobin gene. Other genes or epigenetic modifications may play an important role in HS that were not analyzed in this study.

About 42.4% of HS patients with genotype 2–2 and 25% with genotype 1–2 had a positive family history of HS in our study, compared to 6.7% with genotype 1–1. These results suggest that the Hp‐2 allele may play a substantial role in familial HS. The haptoglobin gene is located on chromosome 16 at locus 22.2 (16q22.2) [35, 36, 37]. To our knowledge, no investigations are currently available on the haptoglobin gene in patients with familial HS. We recommend further investigation of haptoglobin, and especially allele 2, in HS patients in the future.

## 5 Conclusion

For the first time, we performed Hp genotyping in HS patients and found that HS patients with Hp genotype 2–2 are significantly associated with familial HS (42.4%) than the other genotypes (Hp 1–1 and 1–2; 6.7% and 25.0%). Furthermore, HS patients with genotypes 2–2 are associated with more flare‐ups than the other genotypes. Our analysis showed that the distribution of Hp genotypes was similar to the European distribution and our control group. Further research into the haptoglobin gene is recommended to investigate the role of haptoglobin in HS and inflammatory processes.

## Ethics Statement

The study was approved by the Ethics Committee of Ruhr‐University Bochum (protocol code 5076‐14 and date of approval 02 July 2014).

## Consent

Informed consent was obtained from all patients.

## Conflicts of Interest

N.A.R. received funding, travel support, and/or personal honoraria for lectures from Novartis Pharma and Johnson & Johnson independent of the work submitted. Falk G. Bechara has received honoraria for participation in advisory boards, in clinical trials, and/or as a speaker from AbbVie Inc., AbbVie Deutschland GmbH & Co. KG, Acelyrin, Beiersdorf, Boehringer Ingelheim Pharma GmbH & Co. KG, Celltrion, Dr. Wolff, Incyte Corporation, JanssenCilag GmbH, Johnson & Johnson, Merck, Mölnlycke, MoonLake, Novartis Pharma GmbH, Sanofi, Sitala, and UCB Pharma. E.S. has received lecture fees from Almirall, Leo, Pierre Favre, and Philips. L.O. has received honoraria as a speaker and/or travel support from Novartis Pharma GmbH, Incyte Biosciences Corporation, and Janssen. The authors declare no conflicts of interest.

## Data Availability

Data from the study are available upon request from the corresponding author.
